# Impact of three dimensional tomosynthesis on the detection and diagnosis of breast lesions

**DOI:** 10.1186/1470-7330-14-S1-P11

**Published:** 2014-10-09

**Authors:** Sahar Mansour, Lamia Adel, Omina Mokhtar, Omar Sherif Omar

**Affiliations:** 1 Cairo University, Giza, Egypt

## Objective

To evaluate the impact of adding 3D Tomosynthesis to Full Field Digital Mammography (FFDM) in the detection and diagnosis of breast lesions.

## Subjects and methods

The study included 166 mammograms with indeterminate findings selected from 1600 mammograms. They were classified into two groups: group 1 ‘Diagnostic mammograms’ of symptomatic women and group 2 ‘Screening mammograms’. Dense breasts assigned as ACR3 and ACR4 presented 69% (*n* = 114/166) of the studied cases. FFDM and 3D tomosynthesis examination was done and imaging findings were evaluated before and after the use of 3D tomosynthesis images.

## Results

Both modalities were compared regarding detection and diagnosis, each individually assessed, using the Pearson Chi Square tests. Detection (*P* value: 0.006) and diagnosis (*P* value: 0.048) of breast lesions dramatically improved when 3D tomosynthesis images were considered in the evaluation. The sensitivity, specificity, and accuracy of digital mammography was 60%, 20.7% and 48% have significantly enhanced on applying tomosynthesis to be 94.5%, 74% and 89.7%.

## Conclusion

Three-dimensional tomosynthesis significantly enhanced the detection and characterization of breast lesions on digital mammography especially in the context of dense breast parenchyma (ACR 3&4).

